# Associates and predictors of pleurisy or pericarditis in SLE

**DOI:** 10.1136/lupus-2017-000221

**Published:** 2017-10-23

**Authors:** Seungwon Ryu, Wei Fu, Michelle A Petri

**Affiliations:** 1 Konkuk University School of Medicine, Seoul, Republic of Korea; 2 Division of Rheumatology, Johns Hopkins University School of Medicine, Baltimore, Maryland, USA

**Keywords:** systemic lupus erythematosus, serositis, pericarditis, pleurisy

## Abstract

**Background/Purpose:**

Serositis is one of both ACR and SLICC classification criteria for systemic lupus erythematosus (SLE) and a common type of extra renal flare. However, little is known about clinical or immunological associations of pleurisy or pericarditis. The aim of this study is to analyze associates and predictors of pleurisy versus pericarditis in Caucasians and African Americans with SLE.

**Methods:**

2,390 SLE patients in the Hopkins Lupus Cohort were analyzed for demographic, clinical and serologic associates of pleurisy or pericarditis, defined using the SELENA revision of the SLE Disease Activity Index (SLEDAI). The cross-sectional and prospective study using either univariate or multivariate analysis were performed to evaluate the associates of serositis in SLE. We reported associates with a p-value of less than 0.05 for pleurisy or pericarditis.

**Results:**

43% had pleurisy and 22% had pericarditis. African-American ethnicity was a predictive factor for new pericarditis. Hemolytic anemia, proteinuria, lymphadenopathy and anti-Sm were predictive only of pericarditis, whereas pulmonary fibrosis and GI infarction were predictive only of pleurisy. Fever, Raynaud’s syndrome, and anti-DNA were predictors for both pericarditis and pleurisy.

**Conclusion:**

Our study provides further insights into the associates of pleurisy and pericarditis in SLE. Predictors of pleurisy and pericarditis are shown for the first time. The long term consequences from the cross-sectional analysis gives a lesson that serositis in SLE should not be considered benign.

## Introduction 

Both the revised American College of Rheumatology (ACR) and Systemic Lupus International Collaborating Clinics (SLICC) classification criteria for SLE include serositis.^1–3^ Although not among the most common lupus manifestations, serositis is still frequent, seen in 12%–45%.^4–10^ The frequency of pericarditis ranges from 10% to 24%, and of pleurisy ranges from 15% to 34%,^10–15^ suggesting that these two types of serositis have similar frequencies. In one study, 24% of patients with SLE with serositis had relapsed during the follow-up period.^4^

Clinical or immunological associations of pleurisy or pericarditis in SLE are largely unknown.^11–15^ The results from a few past studies have shown some potential associations of pleurisy or pericarditis in SLE, as summarised in table 1.^11–15^ Feng *et al* found that male gender was associated with both serositis (p<0.05) and pleurisy (p<0.05).^11^ In only one study, haemolytic anaemia was associated with pericarditis (p=0.0019) and pleurisy (p=0.028).^12^ Szodoray *et al* found that a low level of vitamin D (p=0.013) was associated with pericarditis.^13^ Tang *et al* showed that anti-Sm (p=0.002) and anti-Jo-1 (p<0.001) were associated with SLE pericarditis.^14^ In one study, pleurisy was associated with younger age at the diagnosis of SLE (p=0.009), higher SLICC/ACR Damage Index (p≤0.0001), greater disease duration (p=0.002), anti-Sm (p=0.002) and anti-RNP (p=0.002).^15^ Although some of these studies included large numbers of patients with SLE, they were usually limited to one ethnicity and failed to confirm previous associations.

**Table 1:** Associations of serositis in SLE

Reference	Ethnicity	Number of patients	Frequency of serositis	Associated factors with pleurisy or pericarditis
Mittoo *et al*^15^	Multiethnic group (distribution: 64.9% Caucasian, 11.8% Asian 8.6% Afro-Caribbean, 5.7% Aboriginal, 4.8% were of other ethnic descent, 4.2% not reported)	876	(Pleurisy) 33.8%	Pleurisy: higher SDI (SLICC/ACR Damage Index Score) (p<0.001), greater disease duration (p=0.002), age at SLE diagnosis (p=0.009), anti-Sm (p=0.002), anti-RNP (p=0.002)
Feng *et al*^11^	Chinese	1790	(Pleurisy) 16.6% (Pericarditis) 9.5%	Pleurisy: male (p<0.05)
Jeffries *et al*^12^	Multiethnic group (distribution: 36.8% European- Americans, 35.5% African- Americans, 15.8% Hispanics, 6.6% Gullah African- Americans, 5.3% American- Indians)	1251	(Pleurisy) 27.4% (Pericarditis) 19.4%	Pleurisy, pericarditis: haemolytic anaemia (p=0.028 for pleurisy, p=0.0019 for pericarditis)
Szodoray *et al*^13^	Hungarian	177	(Pericarditis) 23.7%	Pericarditis: low vitamin D level (p=0.013)
Tang *et al*^14^	Chinese	917	(Pleurisy) 29.7% (Pericarditis) 11.3%	Pericarditis: anti-Sm (p=0.002), anti-Jo-1 (p<0.001)

ACR, American College of Rheumatology; SDI, SLICC/ACR Damage Index; SLICC, Systemic Lupus International Collaborating Clinics.

The Hopkins Lupus Cohort allowed us to study serositis in a large number of patients with SLE. We examined associations of EVER having pleurisy or pericarditis and of developing NEW pleurisy or pericarditis after cohort entry. In particular, we were able to examine associated or predictive factors in both Caucasian and African-American patients, and to find separate associated or predictive factors of pleurisy and pericarditis.

## Methods

### Study group and diagnostic criteria

There were 2390 patients with SLE enrolled in the Hopkins Lupus Cohort at the time of the analysis. The cohort was approved by the Johns Hopkins University School of Medicine Institutional Review Board on a yearly basis. All participants signed informed consent.^16^

We characterised patients with pericarditis or pleurisy, according to the definition of the Safety of Estrogens in Lupus Erythematosus National Assessment–SLE Disease Activity Index (SELENA-SLEDAI).^17^ Pericarditis was diagnosed when the patients had pericardial pain, pericardial friction rub or pericardial effusion or ECG or echocardiogram confirmation of pericarditis. Pleurisy was diagnosed by pleuritic chest pain, pleural rub or pleural effusion. As we used the SLEDAI, we were not able to do separate analysis of pleural rub or effusion.

At cohort entry, basic demographic characteristics (age, age at cohort entry, age at SLE diagnosis, years of education, sex, ethnicity and smoking history), clinical features and serologic data (including lupus anticoagulant, anticardiolipin, Coombs test, anti-dsDNA, anti-La, anti-RNP, anti-Sm, complement C3 and C4 levels and erythrocyte sedimentation rate (ESR)) were collected, and updated at each follow-up visit (at least quarterly).

Organ damage was recorded using the SLICC/ACR Damage Index (SDI).^18^ The index assessed 12 different organs: ocular, neuropsychiatric, renal, pulmonary, cardiovascular, peripheral vascular, gastrointestinal, musculoskeletal system, skin, premature gonadal failure, diabetes and malignancy.

### Statistical analysis

Patients with serositis were compared with patients without serositis, with respect to demographic, clinical and serologic characteristics. For continuous variables (age, age at cohort entry, age at SLE diagnosis, years of education and SDI score), the Student’s t-test was used to compare means. Pearson’s Χ^2^ test was used to analyse categorical variables. Fisher’s exact test was used, instead of Pearson’s, when >20% of the cells in 2×2 contingency table had a frequency <5. The significance level (α) was 0.05 for two-tailed tests. The ORs and 95% CIs were computed to compare the strength of the association.

Cox proportional hazard models were applied to evaluate the association (HR) between demographic characteristics, clinical and serologic features, and newly diagnosed serositis during follow-up. Univariate Cox proportional hazard models were done with adjustment for age and ethnicity. Bonferroni correction was used to account for multiple comparisons in the univariate models. Multivariate Cox proportional hazard models were adjusted for the statistically significant (p<0.0003) covariates from the univariate analysis. The HRs and 95% CIs for each variable were calculated to compare the prognostic strength. All statistical analysis were done with JMP, a statistical software (SAS Institute, Carey, North Carolina, USA).

## Results

### Characteristics of study population

Five hundred and twenty-six patients (22%) had pericarditis and 1037 (43%) had pleurisy. Most were female (94%). Ethnicity was grouped into four categories: 1286 (54%) Caucasians, 923 (39%) African-Americans, 86 (4%) Asians and 94 (4%) other ethnicity.

The revised ACR classification criteria included 49% malar rash, 20% discoid rash, 52% photosensitivity, 52% oral ulcer, 72% arthritis, 48% serositis, 47% renal disorder, 12% neurological disorder, 66% haematological disorder, 81% immunological disorder and 96% ANA positivity. Additional SLICC classification criteria included 20% direct Coombs test, 55% low C3, 48% low C4 and 16% low CH50.

Of the 2390 patients with SLE in the cohort, 2132 patients (89%) were included in the prospective analysis, after excluding those who had serositis before SLE diagnosis. Among the prospective subset, 421 (20%) had pericarditis and the median follow-up time (including censored data) was 106 months (IQR 39.4–191.5). Seven hundred thirty-seven (35%) had new pleurisy and the median follow-up time (including censored data) was 108 months (30.0–167.3).

Associates from cross-sectional study and predictors from prospective study of demographic, clinical, serologic and organ damage-related variables for pericarditis or pleurisy are summarised in tables 2 and 3. Except otherwise noted, only significantly (p<0.0001) associated factors with either pericarditis or pleurisy were mentioned in this section.

**Table 2:** Associates and predictors of pericarditis in SLE among demographic, clinical, serologic features and SDI (p<0.05)

Subgroup	With pericarditis	Without pericarditis	Cross-sectional			Prospective		
			OR	95**%** CI	p Value	HR	95**%** CI	p Value
**Demographic features**								
African-American ethnicity	53.64%	38.54%	1.85	1.51 to 2.26	**<0.0001**	1.91	1.56 to 2.33	**<0.0001**
Private insurance	74.40%	79.45%	0.75	0.60 to 0.95	0.0151	1.01	0.80 to 1.27	0.9656
Age at SLE diagnosis	30.69±12.83	32.90±12.96	0.99	0.98 to 0.99	**0.0006**	0.99	0.98 to 1.00	0.0034
Years of education	14.10±2.85	14.40±3.20	0.97	0.94 to 1	0.0913	1.00	0.96 to 1.03	0.7865
**Clinical features**								
Pulmonary hypertension	17.47%	5.23%	3.83	2.83 to 5.20	**<0.0001**	1.63	0.92 to 2.89	0.0916
Haemolytic anaemia	16.37%	8.34%	2.15	1.61 to 2.87	**<0.0001**	2.34	1.67 to 3.28	**<0.0001**
Fever	50.76%	32.60%	2.13	1.75 to 2.60	**<0.0001**	1.93	1.54 to 2.41	**<0.0001**
Pulmonary fibrosis	13.71%	7.16%	2.06	1.52 to 2.80	**<0.0001**	1.34	0.75 to 2.41	0.327
Nephrotic syndrome	26.78%	15.33%	2.02	1.60 to 2.55	**<0.0001**	1.5	1.13 to 2	0.0053
Proteinuria	57.36%	40.37%	1.99	1.63 to 2.42	**<0.0001**	1.72	1.38 to 2.14	**<0.0001**
Seizure	14.31%	8.23%	1.86	1.39 to 2.50	**<0.0001**	1.13	0.67 to 1.91	0.6393
Haematuria	37.79%	24.78%	1.84	1.50 to 2.26	**<0.0001**	1.73	1.35 to 2.23	**<0.0001**
Lymphadenopathy	41.79%	28.35%	1.81	1.48 to 2.22	**<0.0001**	1.85	1.44 to 2.36	**<0.0001**
Raynaud’s syndrome	63.34%	48.90%	1.81	1.48 to 2.21	**<0.0001**	1.6	1.27 to 2	**0.0001**
Anaemia	71.37%	60.29%	1.64	1.33 to 2.03	**<0.0001**	1.62	1.3 to 2	**<0.0001**
Arthritis	94.67%	90.26%	1.91	1.27 to 2.89	0.0012	1.39	1.13 to 1.71	0.0016
Arthralgia	77.88%	70.40%	1.48	1.18 to 1.86	0.0007	1.45	1.18 to 1.78	**0.0004**
Organic brain syndrome	6.11%	3.61%	1.74	1.26 to 2.68	0.0178	1.42	0.79 to 2.53	0.2392
Pancreatitis	5.16%	3.07%	1.72	1.08 to 2.75	0.0218	1.32	0.59 to 2.96	0.5027
Livedo reticularis	22.69%	28.31%	0.74	0.59 to 0.93	0.0108	0.91	0.62 to 1.32	0.6046
**Serologic features**								
ESR	83.85%	71.71%	2.05	1.59 to 2.64	**<0.0001**	1.14	0.9 to 1.45	0.2898
Anti-DNA	73.09%	58.74%	1.91	1.54 to 2.36	**<0.0001**	1.73	1.41 to 2.12	**<0.0001**
Anti-Sm	28.80%	17.97%	1.85	1.47 to 2.32	**<0.0001**	1.72	1.32 to 2.24	**0.0001**
Low C3	65.46%	51.86%	1.76	1.44 to 2.15	**<0.0001**	1.56	1.24 to 1.97	**0.0002**
Low C4	57.63%	44.85%	1.67	1.38 to 2.04	**<0.0001**	1.64	1.29 to 2.08	**<0.0001**
Anti-RNP	37.82%	25.89%	1.74	1.41 to 2.14	**<0.0001**	1.3	0.95 to 1.77	0.0962
PLT <100	26.58%	18.83%	1.56	1.24 to 1.96	**0.0002**	1.28	0.92 to 1.78	0.1505
Coombs test	25.29%	18.79%	1.46	1.14 to 1.88	0.0036	1.5	1.09 to 2.06	0.0134
Leucopenia	50.38%	44.39%	1.27	1.05 to 1.54	0.0171	1.41	1.13 to 1.77	0.0028
**SDI**								
**Total SDI score**	3.08±2.78	1.85±2.32						
*Pulmonary*	0.30±0.62	0.12±0.38						
Pulmonary hypertension	10.33%	3.21%	3.48	2.37 to 5.10	**<0.0001**	1.45	0.64 to 3.29	0.3723
Pleural fibrosis	5.57%	2.02%	2.86	1.74 to 4.70	**<0.0001**	1.04	0.26 to 4.19	0.9604
Pulmonary fibrosis	12.43%	5.92%	2.26	1.63 to 3.12	**<0.0001**	1.1	0.54 to 2.24	0.7879
Shrinking lung	0.96%	0.16%	5.9	1.41 to 24.79	0.0161	3.35	0.47 to 23.96	0.2285
*Cardiovascular*	0.33±0.67	0.12±0.44						
Cardiomyopathy	8.62%	2.45%	3.76	2.46 to 5.76	**<0.0001**	1.45	0.59 to 3.55	0.4153
Valvular disease	5%	2.07%	2.5	1.50 to 4.15	**0.0003**	0.9	0.22 to 3.63	0.8814
Myocardial infarction ever	6.7%	3.7%	1.87	1.23 to 2.85	0.0030	1.85	0.8 to 4.27	0.1493
*Gastrointestinal*	0.28±0.58	0.14±0.42						
Infarction or resection of bowel	20.65%	11.24%	2.06	1.59 to 2.66	**<0.0001**	1.98	1.28 to 3.05	0.0020
*Musculoskeletal*	0.59±0.96	0.36±0.74						
Deforming or erosive arthritis	9.75%	5.4%	1.89	1.33 to 2.70	**0.0003**	1.03	0.52 to 2.02	0.9375
Osteoporosis	16.63%	10.75%	1.66	1.26 to 2.18	**0.0003**	0.54	0.24 to 1.24	0.1468
Avascular necrosis	15.49%	9.67%	1.71	1.29 to 2.27	**0.0002**	0.91	0.52 to 1.57	0.7340
*Renal*	0.40±1.05	0.22±0.78						
Renal insufficiency	9.96%	5.17%	2.03	1.43 to 2.89	**<0.0001**	1.73	1.08 to 2.76	0.0218
Proteinuria	11.88%	6.92%	1.81	1.32 to 2.50	**0.0002**	1.44	0.95 to 2.18	0.0825
End-stage renal disease	7.66%	4.14%	1.92	1.29 to 2.86	0.0010	1.23	0.61 to 2.51	0.5631
*Ocular*	0.26±0.49	0.20±0.46						
Any cataract ever	21.22%	15.86%	1.43	1.12 to 1.82	0.0040	0.95	0.57 to 1.59	0.8489
*Malignancy*	13.63%	9.83%	1.45	1.08 to 1.94	0.0131	1.74	0.93 to 3.26	0.0818
*Peripheral vascular*	0.10±0.36	0.06±0.28						
Venous thrombosis	6.13%	3.04%	2.08	1.33 to 3.25	0.0010	1.12	0.46 to 2.73	0.7989
Significant tissue loss ever	1.92%	0.76%	2.55	1.23 to 5.78	0.0200	2.8	1.03 to 7.57	0.0432
*Neuropsychiatric*	0.38±0.72	0.30±0.64						
Seizures	6.7%	4.02%	1.72	1.13 to 2.60	0.0099	0.82	0.34 to 2	0.6689
*Diabetes*	10.38%	7.4%	1.45	1.04 to 2.02	0.0275	0.97	0.48 to 1.99	0.9388
*Premature gonadal failure*	6.54%	4.3%	1.56	1.03 to 2.36	0.0347	1.35	0.66 to 2.76	0.4096

*After adjusting for multiple comparisons, significant level is 0.0006; p<0.0006 bold.

ACR, American College of Rheumatology; ESR, erythrocyte sedimentation rate; SDI, **SLICC/ACR Damage Index; **SLICC, Systemic Lupus International Collaborating Clinics; PLT, platelet.

**Table 3:** Associates and predictors of pleurisy in SLE among demographic, clinical, serologic features and SDI (p<0.05)

Subgroup	With pleurisy	Without pleurisy	Cross-sectional			Prospective		
			OR	95% CI	p Value	HR	95% CI	p Value
**Demographic features**								
Age at SLE diagnosis	31.03±12.01	33.44±13.54	0.99	0.98 to 0.99	**<0.0001**	0.99	0.98 to 1.00	**0.0002**
African-American ethnicity	44.04%	40.03%	1.17	0.99 to 1.40	0.0615	0.92	0.66 to 1.28	0.6299
**Clinical features**								
Arthralgia	95.85%	87.66%	3.25	2.30 to 4.60	**<0.0001**	1.32	1.13 to 1.54	**0.0005**
Pancreatitis	5.50%	2.00%	2.86	1.79 to 4.55	**<0.0001**	3.25	1.91 to 5.54	**<0.0001**
Pulmonary fibrosis	12.17%	5.76%	2.27	1.69 to 3.04	**<0.0001**	2.2	1.41 to 3.43	**0.0005**
Fever	45.95%	29.39%	2.04	1.72 to 2.42	**<0.0001**	1.87	1.56 to 2.23	**<0.0001**
Arthritis	79.48%	66.32%	1.97	1.63 to 2.37	**<0.0001**	1.38	1.18 to 1.62	**<0.0001**
Pulmonary hypertension	10.57%	5.78%	1.93	1.42 to 2.61	**<0.0001**	0.96	0.54 to 1.71	0.8805
Haemolytic anaemia	12.87%	7.91%	1.72	1.31 to 2.26	**<0.0001**	1.51	1.1 to 2.08	0.0106
Lymphadenopathy	37.78%	26.34%	1.7	1.43 to 2.02	**<0.0001**	1.53	1.24 to 1.89	**0.0001**
Anaemia	69.57%	57.51%	1.69	1.42 to 2.00	**<0.0001**	1.46	1.23 to 1.72	**<0.0001**
Raynaud’s syndrome	58.37%	47.08%	1.58	1.34 to 1.86	**<0.0001**	1.55	1.3 to 1.84	**<0.0001**
Malar rash	54.26%	45.75%	1.41	1.20 to 1.65	**<0.0001**	1.24	1.05 to 1.46	0.0112
Proteinuria	48.64%	40.70%	1.38	1.17 to 1.63	**0.0001**	1.24	1.04 to 1.49	0.0168
Mouth ulcer	55.66%	48.48%	1.33	1.13 to 1.57	**0.0005**	1.34	1.12 to 1.6	0.0013
Alopecia	58.00%	52.14%	1.27	1.08 to 1.49	0.0047	1.12	0.94 to 1.34	0.2066
Nephrotic syndrome	20.20%	16.08%	1.32	1.07 to 1.63	0.0107	1.2	0.94 to 1.53	0.1489
Haematuria	30.23%	25.65%	1.26	1.05 to 1.50	0.0133	1.32	1.07 to 1.63	0.0100
Seizure	11.00%	8.43%	1.34	1.02 to 1.77	0.0352	1.02	0.67 to 1.55	0.9419
Photosensitivity	54.70%	50.48%	1.18	1.01 to 1.39	0.0427	1.21	1.01 to 1.44	0.0356
Organic brain syndrome	5.12%	3.41%	1.53	1.02 to 2.29	0.0486	1.36	0.84 to 2.21	0.2112
**Serologic features**								
ESR	79.51%	70.40%	1.63	1.35 to 1.98	**<0.0001**	1.15	0.95 to 1.38	0.1592
Anti-DNA	66.99%	58.01%	1.47	1.24 to 1.74	**<0.0001**	1.55	1.33 to 1.81	**<0.0001**
Low C3	60.10%	50.81%	1.46	1.24 to 1.72	**<0.0001**	1.48	1.23 to 1.78	**<0.0001**
Low C4	52.75%	43.77%	1.43	1.22 to 1.69	**<0.0001**	1.59	1.32 to 1.91	**<0.0001**
Coombs test	23.66%	17.53%	1.46	1.17 to 1.82	0.0009	1.43	1.1 to 1.87	0.0086
Anti-RNP	31.41%	26.34%	1.28	1.07 to 1.54	0.0081	1.19	0.92 to 1.53	0.1864
Anti-Sm	22.56%	18.78%	1.26	1.03 to 1.54	0.0255	1.58	1.27 to 1.96	**<0.0001**
**SDI**								
**Total SDI score**	2.63±2.73	1.73±2.20						
*Pulmonary*	0.22±0.53	0.11±0.38						
Pulmonary hypertension	6.41%	3.44%	1.92	1.31 to 2.83	0.0007	1.35	0.67 to 2.73	0.4021
Pleural fibrosis	3.89%	1.95%	2.03	1.23 to 3.35	0.0047	0.32	0.04 to 2.27	0.2538
*Cardiovascular*	0.23±0.58	0.12±0.43						
Pericarditis	3.11%	0.67%	4.74	2.25 to 9.97	**<0.0001**	2.02	1 to 4.07	0.0485
Myocardial infarction ever	5.92%	3.14%	1.94	1.30 to 2.91	0.0010	0.71	0.26 to 1.93	0.5043
Cardiomyopathy	5.15%	2.76%	1.91	1.24 to 2.93	0.0026	1.54	0.79 to 3	0.2028
Valvular disease	3.60%	2.02%	1.81	1.10 to 2.99	0.0190	2.16	0.89 to 5.24	0.0897
*Gastrointestinal*	0.23±0.52	0.13±0.40						
Infarction or resection of bowel	17.26%	10.22%	1.83	1.44 to 2.33	**<0.0001**	1.91	1.33 to 2.74	**0.0004**
Chronic peritonitis	0.68%	0.07%	9.17	1.13 to 74.64	0.0247	3.37	0.83 to 13.7	0.0896
*Musculoskeletal*	0.52±0.90	0.33±0.70						
Deforming or erosive arthritis	8.80%	4.38%	2.1	1.50 to 2.96	**<0.0001**	1.02	0.53 to 1.99	0.9464
Osteoporosis	14.81%	9.94%	1.58	1.23 to 2.02	**0.0003**	1.26	0.76 to 2.07	0.3686
Avascular necrosis	13.35%	9.06%	1.55	1.19 to 2.00	0.0009	0.94	0.6 to 1.49	0.7999
*Neuropsychiatric*	0.37±0.72	0.27±0.60						
Cranial or peripheral neuropathy	11.75%	7.47%	1.65	1.25 to 2.18	**0.0004**	0.91	0.52 to 1.59	0.7502
Cerebrovascular accident ever	9.71%	6.95%	1.44	1.07 to 1.93	0.0148	1.2	0.75 to 1.93	0.4526
*Premature gonadal failure*	6.44%	3.58%	1.85	1.27 to 2.71	0.0013	1.47	0.8 to 2.69	0.2160
*Ocular*	0.25±0.48	0.19±0.45						
Any cataract ever	19.75%	14.88%	1.41	1.13 to 1.74	0.0018	0.71	0.43 to 1.17	0.1808
*Diabetes*	9.46%	7.02%	1.38	1.03 to 1.86	0.0308	0.87	0.43 to 1.77	0.7094
*Peripheral vascular*								
Venous thrombosis	5.05%	2.76%	1.87	1.22 to 2.88	0.0037	1.07	0.55 to 2.08	0.8342
*Skin*								
Skin ulceration for >6 months	2.05%	0.75%	2.77	1.30 to 5.91	0.0060	1.99	0.63 to 6.25	0.2410
*Renal*								
Renal insufficiency	7.38%	5.31%	1.42	1.02 to 1.96	0.0383	1.09	0.69 to 1.73	0.7093

*After adjusting for multiple comparisons, significant level is 0.0006; p<0.0006 bold.

ACR, American College of Rheumatology; ESR, erythrocyte sedimentation rate; SDI, **SLICC/ACR Damage Index; **SLICC, Systemic Lupus International Collaborating Clinics.

### Cross-sectional study: association of demographic characteristics with serositis in SLE

African-American ethnicity was highly associated only with pericarditis, and age at SLE diagnosis was significantly associated only with pleurisy. There was no demographic factor that was significantly associated with both pericarditis and pleurisy.

Private health insurance (OR 0.75, p=0.0151) was the only protective factor against pericarditis among demographic characteristics. Gender, age at cohort entry and smoking history were not associated with either pleurisy or pericarditis.

### Cross-sectional study: association of clinical features with serositis in SLE

Factors significantly associated only with pericarditis were nephrotic syndrome, proteinuria, seizure and haematuria. Arthralgia, pancreatitis, arthritis and malar rash were only significantly associated with pleurisy. Factors highly associated with both pleurisy and pericarditis were pulmonary hypertension, fever, pulmonary fibrosis, haemolytic anaemia, lymphadenopathy, Raynaud’s syndrome and anaemia.

### Cross-sectional study: association of serologic features with serositis in SLE

Only pericarditis was highly associated with anti-Sm and anti-RNP. Serologic tests significantly associated with both pericarditis and pleurisy were high ESR, anti-DNA, low C3 and low C4.

### Cross-sectional study: association of organ damage with serositis in SLE

Organ damage that was significantly associated with only pericarditis included renal insufficiency, pulmonary hypertension, pleural fibrosis and cardiomyopathy. Deforming or erosive arthritis was associated only with pleurisy. Pulmonary fibrosis, and gastrointestinal infarction or resection were highly associated with both pleurisy and pericarditis.

### Prospective study: predictive factors for serositis in patients with SLE

A prospective analysis using univariate and multivariate Cox proportional hazard model was done to investigate predictive factors for new serositis in patients with SLE.

The univariate Cox regression model was adjusted for age and ethnicity. The results are shown in the right column of the tables 2 and 3. The analysis of either age or ethnicity for the univariate model was done independently, so as not to miss their own predictive value. For pericarditis, compared with Caucasians, the HR for African-Americans was 1.91 (95% CI 1.56 to 2.33, p<0.0001) and the HR for 1 year increase in age is 0.99 (95% CI 0.98 to 1.00, p=0.0033). For pleurisy, compared with Caucasians, the HR for African-Americans was 1.32 (95% CI 1.13 to 1.53, p=0.0003) and the HR for 1 year increase was 0.99 (95% CI 0.98 to 0.99, p=0.0002).

The results of the multivariate Cox regression (p<0.05) using only covariates from the univariate analysis are shown in tables 4 and 5. African-American, haemolytic anaemia, proteinuria, lymphadenopathy and anti-Sm were predictive factors only for pericarditis. Arthritis, pulmonary fibrosis, low C4 and infarction or resection of bowel were predictors of only pleurisy. Predictors of both pericarditis and pleurisy were fever, Raynaud’s syndrome and anti-DNA.

**Table 4:** Multivariable analysis of pericarditis in SLE (p<0.05)

Clinical features	Cross-sectional			Prospective		
	OR	95% CI	p Value	HR	95% CI	p Value
Age at SLE diagnosis	0.99	0.98 to 0.99	0.0023			
African-American	1.52	1.21 to 1.91	0.0003	1.68	1.38 to 2.05	<0.0001
Haemolytic anaemia				1.76	1.25 to 2.48	0.0014
Fever	1.49	1.19 to 1.87	0.0005	1.5	1.18 to 1.9	0.0009
Proteinuria				1.39	1.11 to 1.74	0.0037
Lymphadenopathy	1.32	1.05 to 1.66	0.0181	1.34	1.03 to 1.74	0.0312
Raynaud’s syndrome	1.62	1.29 to 2.02	<0.0001	1.4	1.11 to 1.77	0.0045
Anti-DNA	1.34	1.05 to 1.72	0.02	1.41	1.13 to 1.75	0.0019
Anti-Sm	1.39	1.08 to 1.79	0.0117	1.33	1.01 to 1.76	0.0417
Pulmonary hypertension	2.65	1.86 to 3.78	<0.0001			
Nephrotic syndrome	1.36	1.04 to 1.77	0.0246			
Seizure	1.69	1.22 to 2.34	0.0015			
Pulmonary fibrosis	1.5	1.02 to 2.2	0.0404			
Pleural fibrosis	2.65	1.49 to 4.71	0.0009			
Cardiomyopathy	2.38	1.42 to 3.99	0.001			
Infarction or resection of bowel	1.93	1.52 to 2.45	<0.0001			

*Logistic regression also adjusts for ESR, which are not shown in the table because association is not statistically significant.

†Cox regression was also adjusted for arthritis, which is not shown in the table as it was not statistically significant.

‡Blank cell indicates the clinical feature was not selected into corresponding multivariate analysis.

**Table 5:** Multivariable analysis of pleurisy in SLE (p<0.05)

Clinical features	Cross-sectional			Prospective		
	OR	95% CI	p Value	HR	95% CI	p Value
Age at SLE diagnosis	0.99	0.98 to 0.99	0.0001			
Arthritis	1.74	1.42 to 2.12	<0.0001	1.36	1.16 to 1.58	0.0001
Pancreatitis	2.12	1.27 to 3.53	0.0039			
Pulmonary fibrosis	1.91	1.39 to 2.61	0.0001	1.63	1.1 to 2.42	0.0157
Fever	1.54	1.28 to 1.85	<0.0001	1.38	1.18 to 1.62	<0.0001
Lymphadenopathy	1.27	1.05 to 1.53	0.0148			
Raynaud’s syndrome	1.29	1.08 to 1.54	0.0051	1.21	1.04 to 1.4	0.0134
Anti-DNA				1.18	1.01 to 1.39	0.0424
Low C3	1.23	1.02 to 1.47	0.0273			
Low C4				1.23	1.01 to 1.48	0.0358
Erythrocyte sedimentation rate	1.26	1.02 to 1.55	0.0300			
Chronic pericarditis or pericardiectomy	3.22	1.56 to 6.63	0.0015			
Infarction or resection of bowel	1.49	1.2 to 1.86	0.0004	1.56	1.21 to 2.0	0.0006

*Logistic regression also adjusts for mouth ulcer, pulmonary hypertension, cranial or peripheral neuropathy, which are not shown in the table because association is not statistically significant,

†Cox regression was also adjusted for pancreatitis, anti-Sm, which are not shown in the table as they were not statistically significant.

‡Blank cell indicates the clinical feature was not selected into corresponding multivariate analysis.

## Discussion

Although cross-sectional analyses of serositis have been done in SLE, ours is the first prospective analysis of new serositis and the first study to emphasise the different associates and predictors of pericarditis versus pleurisy.

It is important to compare the results between the cross-sectional analysis and prospective analyses. The cross-sectional analysis includes all patients, and thus has greater statistical power. The prospective analysis includes only those with new serositis during prospective cohort follow-up. It has fewer numbers, but, as it is prospective, has complete data on predictors that would not have been available from medical records.

### Demographic associates/predictors of serositis in SLE

African-American ethnicity was a predictive factor for new pericarditis (and ever having pericarditis), but not pleurisy. A previous study on a multiethnic Canadian cohort^15^ (64.9% Caucasians, 11.8% Asian, 8.6% Afro-Caribbean, 5.7% Aboriginal, etc) also found no association of pleurisy with ethnicity. Alarcon *et al*,^19^ who included patients from the Hopkins cohort, had previously reported an African-American association with serositis as a whole. A Latin-American study with 1437 multiethnicity patients^20^ reported that ‘primary cardiac disease’, including pericarditis (81.2% of all cases), was associated with African-Latin American ethnicity. Because serositis had been thought to be one pathogenic mechanism, it is quite surprising (and not understood) why pericarditis is associated with African-American ethnicity, whereas pleurisy is not.

Gender was not associated with either pericarditis or pleurisy in either cross-sectional or prospective analysis. In contrast, a Chinese cohort^11^ found an association with male gender, but only in the age group of 15–50 years. We did not have enough Asian patients to do a subset analysis.

Our cross-sectional analysis showed that younger age at SLE diagnosis was associated with both pericarditis and pleurisy. A Canadian, multiethnic study^15^ also found pleurisy to be associated with younger age at SLE diagnosis. A Chinese cohort,^11^ however, found no association of age at SLE onset with either pericarditis or pleurisy. It should be noted that younger age at SLE diagnosis was not predictive of either new pericarditis and pleurisy in our prospective analysis.

Smoking history was not associated with either pericarditis or pleurisy, consistent with one previous report.^15^ However, one study^21^ of 119 patients did find an association with current smoking. The study by Rubin *et al*,^21^ however, only included patients <1 year after diagnosis, and female patients with no history of therapy.

We found that only few variables were shown in the result of multiple variable analysis (tables 4 and 5): age at SLE diagnosis (pericarditis and pleurisy: cross-sectional study) and African-American (pericarditis: cross-sectional and prospective study).

### Clinical associates/predictors of serositis in SLE

We found that haemolytic anaemia was associated with both pericarditis and pleurisy, consistent with the previous result of a multiethnic group study^12^ of 1251 patients. Haemolytic anaemia was predictive of only pericarditis in the multivariate prospective analysis.

According to our univariate cross-sectional analysis, livedo reticularis was a protective factor only for pericarditis (OR=0.74, p=0.0108). Livedo reticularis is commonly found in patients with SLE with antiphospholipid antibodies.^22^ However, in our study, lupus anticoagulant was not protective against pericarditis. Anticardiolipin was also not protective against pericarditis or pleurisy, similar to a Chinese study.^14^ Therefore, it is highly unlikely that the protective association with livedo reticularis was explained by a confounding effect of antiphospholipid antibodies. The protective mechanism of livedo reticularis for pericarditis is not understood.

Central nervous system (CNS) involvement was reported to be a protective factor (OR=0.52, p=0.0302) for pulmonary manifestations, including pleural effusion, in a Latin American study.^20^ We, however, could not find any prospective association of CNS involvement with pericarditis or pleurisy in any of our analysis.

Only three clinical factors (proteinuria, lymphadenopathy and Raynaud’s syndrome) among variables in tables 2 and 3 were associates and predictors for both pericarditis and pleurisy in the multiple variable analysis (tables 4 and 5).

### Serologic associates/predictors of serositis in SLE

The association of anti-DNA with both pericarditis and pleurisy in the univariate cross-sectional and prospective analyses in our study was in contradiction to a previous Chinese cohort study of 917 patients.^14^ Pleural effusion was shown to be associated with anti-DNA in one previous study from Saudi Arabia of 61 patients.^23^

Low complement (C3 and C4) was associated with both pericarditis and pleurisy in our univariate cross-sectional and prospective analyses. Other investigators^24^ observed a relatively weak association of pericardial tamponade with low C4 (p=0.05), compared with pericardial effusions as a whole. In terms of pleurisy, in a Saudi Arabian cohort of 61 patients,^23^ pleural effusion was associated with both low C3 and C4.

ESR was associated with both pericarditis and pleurisy in the univariate cross-sectional analysis. Previously, using the Hopkins Lupus Cohort, we^25^ reported that ESR was correlated with serositis. We now can extend this previous result to an association with both pericarditis and pleurisy individually.

Anti-RNP was associated with both pericarditis and pleurisy in cross-sectional univariate analyses, but not the prospective analysis. An association with pleurisy was shown in the Canadian cohort,^15^ but not in a Chinese cohort of 917 patients.^14^

In our univariate analysis, anti-Sm was associates and predictor of pericarditis and pleurisy, in contrast to a previous report.^14^ Anti-Sm was a predictive factor for only pericarditis in the multiple variable model, as well. The association of anti-Sm with pleurisy was, however, supported by the study of the Canadian cohort.^15^

Our multivariable analysis (tables 4 and 5) for serologic variables showed that anti-DNA and anti-Sm were both associates and predictors for pericarditis. They were factors shown in only one study for pleurisy: anti-DNA (prospective), low C4 (prospective) and low C3 (cross-sectional study).

### Damage associates/predictors of serositis in SLE

Permanent organ damage was assessed by SDI. The multivariable, prospective study showed that pleurisy predicted later gastrointestinal infarction or resection, but there was no factor predictive of pericarditis.

### Conclusion

Our study shows, for the first time, the different predictors of pleurisy and pericarditis in SLE. The cross-sectional analysis also defines the long-term consequences, especially of pulmonary fibrosis and infarction of bowel. Serositis in SLE should not be considered benign, as it was associated with long-term organ damage in SLE.

### Strengths and limitations

The strengths of our study are the large number of patients, the ethnic diversity and the cohort structure with quarterly follow-up and protocolised laboratory assessment. Predictors from the prospective analysis are anticipated to give the most important information for clinicians. We are the first to separately analyse associates and predictors of pleurisy versus pericarditis.

This study still has several limitations. We did not use cardiac ultrasound or chest X-ray when the clinical diagnosis of serositis was secure. Our results may not be generalisable to the most severe forms of serositis in patients with SLE. To avoid the issue of multiple comparisons, we have emphasised variables that met the most stringent p value cut-off, and thus may have missed some borderline associations.
